# Computational translation of genomic responses from experimental model systems to humans

**DOI:** 10.1371/journal.pcbi.1006286

**Published:** 2019-01-10

**Authors:** Douglas K. Brubaker, Elizabeth A. Proctor, Kevin M. Haigis, Douglas A. Lauffenburger

**Affiliations:** 1 Department of Biological Engineering, Massachusetts Institute of Technology, Cambridge, MA, United States of America; 2 Cancer Research Institute, Beth Israel Deaconess Cancer Center and Department of Medicine, Harvard University Medical School, Boston, MA, United States of America; 3 Departments of Neurosurgery and Pharmacology, Penn State College of Medicine, Hershey, Pennsylvania, United States of America; 4 Department of Biomedical Engineering, Pennsylvania State University, State College, Pennsylvania, United States of America; NYU, UNITED STATES

## Abstract

The high failure rate of therapeutics showing promise in mouse models to translate to patients is a pressing challenge in biomedical science. Though retrospective studies have examined the fidelity of mouse models to their respective human conditions, approaches for prospective translation of insights from mouse models to patients remain relatively unexplored. Here, we develop a semi-supervised learning approach for inference of disease-associated human differentially expressed genes and pathways from mouse model experiments. We examined 36 transcriptomic case studies where comparable phenotypes were available for mouse and human inflammatory diseases and assessed multiple computational approaches for inferring human biology from mouse datasets. We found that semi-supervised training of a neural network identified significantly more true human biological associations than interpreting mouse experiments directly. Evaluating the experimental design of mouse experiments where our model was most successful revealed principles of experimental design that may improve translational performance. Our study shows that when prospectively evaluating biological associations in mouse studies, semi-supervised learning approaches, combining mouse and human data for biological inference, provide the most accurate assessment of human *in vivo* disease processes. Finally, we proffer a delineation of four categories of model system-to-human “Translation Problems” defined by the resolution and coverage of the datasets available for molecular insight translation and suggest that the task of translating insights from model systems to human disease contexts may be better accomplished by a combination of translation-minded experimental design and computational approaches.

## Author summary

Empirical comparison of genomic responses in mouse models and human disease contexts is not sufficient for addressing the challenge of prospective translation from mouse models to human disease contexts. We address this challenge by developing a semi-supervised machine learning approach that combines supervised modeling of mouse datasets with unsupervised modeling of human disease-context datasets to predict human *in vivo* differentially expressed genes and enriched pathways. Semi-supervised training of a feed forward neural network was the most efficacious model for translating experimentally derived mouse biological associations to the human *in vivo* disease context. We find that computational generalization of signaling insights substantially improves upon direct generalization of mouse experimental insights and argue that such approaches can facilitate more clinically impactful translation of insights from preclinical studies in model systems to patients.

## Introduction

Generalization of insights from disease model systems to the human *in vivo* context remains a persistent challenge in biomedical science. The association of molecular features with a phenotype in a model system often does not hold true in the corresponding human indication, due to some combination of the fidelity of the experimental system to human *in vivo* biology and the inherent complexity of human disorders [1–7]. Though it is now routine to collect clinical samples from patients and associate molecular features with clinical phenotypes, there are discrepancies between the phenotypes measurable in patients and those investigable by use of model systems. Outside of a clinical trial, novel perturbations to the disease system cannot be directly investigated in the patient *in vivo* context, whereas model systems can be used to study the impact of innumerable perturbations to the disease system and to associate molecular features with these responses. As a consequence of this discrepancy, murine and other model systems of disease are likely to remain an important part of biomedical research. Therefore, methods for improving generalizability of mouse-derived molecular signatures to human *in vivo* contexts are needed for more impactful translational research.

The utility of mouse models for studying inflammatory pathologies was recently assessed by a pair of studies examining the correspondence between gene expression in murine models of inflammatory pathologies and human contexts [1, 2]. In these studies, mouse molecular and phenotype data were matched to human *in vivo* molecular and phenotype data, enabling direct comparison of genomic responses between mice and humans. These studies analyzed the same datasets and came to conflicting conclusions about the relevance of mouse models for inflammatory disease research, with Seok *et al*. concluding that mouse models poorly mimic human pathologies and Takao *et al*. concluding that mouse models usefully mimic human pathologies [1, 2]. A key methodological difference between the two studies was that Takao *et al*. examined genes significantly changed in both contexts [1, 2]. However, in prospective translational studies, the corresponding mouse and human *in vivo* datasets and perturbations are rarely available making accurate pre-selection of genes that change in both human and mouse contexts unlikely. Therefore, prospective studies will often need to proceed on the basis of molecular changes in the model system alone.

The aim of our study is to develop a machine learning approach to address the challenge of prospective inference of human biology from model systems. Here, we consider a machine learning approach successful if it correctly predicts a higher proportion of human differentially expressed genes (DEG) and enriched signaling pathways than implicated by the corresponding mouse model. The essence of our approach is to apply a machine learning classifier to assign predicted phenotypes, derived from a mouse dataset, to molecular datasets of disease-context human samples and to infer human DEGs and enriched pathways downstream of the machine learning model using these inferred phenotypes. We assessed our approach by testing it on the datasets from the Seok and Takao studies, where mouse phenotypes and gene expression data were matched to patient clinical phenotypes and gene expression data [1, 2, 8–20].

While mouse experiments alone failed to capture a large portion of human *in vivo* biology, using these datasets to train computational models produced more precise and comprehensive predictions of human *in vivo* biology. In particular, semi-supervised training of a neural network identified significantly more human *in vivo* DEGs and pathways than mouse models alone or other machine learning approaches examined here. We identify aspects of model system study design that influence the performance of our neural network and show that the added benefit of our method is driven by recovery of biological processes not present in the mouse disease models. Our results suggest that computational generalization of insights from mouse model systems better predicts human *in vivo* disease biology and that such approaches may facilitate more clinically impactful translation of model system insights.

## Results

### Developing a framework for mouse-to-human genomic insight translation

We assembled a cohort of mouse-to-human translation case studies from the datasets analyzed in Seok *et al*. and Takao *et al*. (Table 1) [1] [2]. We defined case studies as all pairs of mouse (training dataset) and human (test dataset) datasets for the same disease condition. By constructing case studies in this manner, multiple mouse strains and experimental protocols could be compared to different presentations of that same disease in independent human cohorts. The final cohort consisted of 36 mouse-to-human translation case studies in which mouse-to-human biological correspondence and machine learning translation approaches could be assessed (Table 2).

**Table 1:** Cohort of mouse and human inflammatory pathology microarray datasets. Datasets are identified by Gene Expression Omnibus (GEO) accession numbers and microarray platorms. Conditions, sample sizes, and tissue source for each dataset are shown. Inflammatory disease inductions include lipopolysaccharide (LPS), cecal ligation and puncture (CLP), Streptococcus Pneumoniae Serotype 2 (SPS2), and Staphylococcus Aureus (SA) exposure.

Species	Disease	GEO Accession	Microarray Platform(s)	Conditions and Sample Sizes	Tissue
Human	Burn	GSE37069	GPL570	Control (n = 37) Burn (n = 553)	White Blood Cells
Human	Trauma	GSE36809	GPL570	Control (n = 37) Trauma (n = 216)	White Blood Cells
Human	Endotoxemia	GSE3284	GPL96 GPL 97	Control (n = 16) Endotoxin (n = 76)	Whole Blood
Human	Sepsis	GSE13904	GPL570	Control (n = 18) Sepsis (n = 158)	Whole Blood
Human	Sepsis	GSE9960	GPL570	Control (n = 16) Sepsis (n = 54)	Peripheral Blood Mononuclear Cells
Human	Sepsis	GSE28750	GPL570	Control (n = 20) Sepsis (n = 21)	Whole Blood
Human	Sepsis	GSE13015	GPL6106 GPL6947	Control (n = 29) Sepsis (n = 77)	Whole Blood
Mouse (C57BL/6J)	Burn	GSE7404	GPL1261	Control (n = 16) Burn (n = 16)	Leukocytes
Mouse (C57BL/6J)	Trauma	GSE7404	GPL1261	Control (n = 16) Trauma (n = 16)	Leukocytes
Mouse (C57BL/6J)	Endotoxemia: LPS	GSE7404	GPL1261	Control (n = 8) Endotoxemia (n = 8)	Leukocytes
Mouse (C57BL/6J)	Endotoxemia: LPS	GSE5663	GPL81	Control (n = 4) Endotoxemia (n = 5)	Blood
Mouse (C57BL/6J)	Sepsis: CLP	GSE5663	GPL81	Control (n = 4) Mild Sepsis (n = 5) Sepsis (n = 5)	Blood
Mouse (C57BL/6J)	Sepsis: SPS2	GSE26472	GPL6778	Control (n = 3) Sepsis (n = 8)	Blood
Mouse (A/J and C57BL/6J)	Sepsis: SA	GSE19668	GPL1261	Control A/J (n = 5) Control C57BL6J (n = 5) Sepsis A/J (n = 20) Sepsis C57BL6J (n = 20)	Blood

**Table 2:** Enumeration of mouse-to-human translation case studies. Case studies are defined by all combinations of mouse and human datasets (GEO-GSE number) for a given mouse strain, microarray platform (GPL number), and disease induction method.

Disease Case Study	Mouse (Training Set) GEO Accession	Human (Test Set) GEO Accession
1. Burn	GSE7404	GSE37069
2. Trauma	GSE7404	GSE36809
3. Endotoxemia	GSE7404	GSE3284 (GPL96)
4. Endotoxemia	GSE5663	GSE3284 (GPL96)
5. Endotoxemia	GSE7404	GSE3284 (GPL97)
6. Endotoxemia	GSE5663	GSE3284 (GPL97)
7. Sepsis	GSE5663 (Mild CLP Sepsis)	GSE13904
8. Sepsis	GSE5663 (Mild CLP Sepsis)	GSE9960
9. Sepsis	GSE5663 (Mild CLP Sepsis)	GSE28750
10. Sepsis	GSE5663 (Mild CLP Sepsis)	GSE13015 (GPL6947)
11. Sepsis	GSE5663 (Mild CLP Sepsis)	GSE13015 (GPL6106)
12. Sepsis	GSE5663 (CLP Sepsis)	GSE13904
13. Sepsis	GSE5663 (CLP Sepsis)	GSE9960
14. Sepsis	GSE5663 (CLP Sepsis)	GSE28750
15. Sepsis	GSE5663 (CLP Sepsis)	GSE13015 (GPL6947)
16. Sepsis	GSE5663 (CLP Sepsis)	GSE13015 (GPL6106)
17. Sepsis	GSE26472 (SPS2 Sepsis)	GSE13904
18. Sepsis	GSE26472 (SPS2 Sepsis)	GSE9960
19. Sepsis	GSE26472 (SPS2 Sepsis)	GSE28750
20. Sepsis	GSE26472 (SPS2 Sepsis)	GSE13015 (GPL6947)
21. Sepsis	GSE26472 (SPS2 Sepsis)	GSE13015 (GPL6106)
22. Sepsis	GSE19668 (AJ Strain, SA)	GSE13904
23. Sepsis	GSE19668 (AJ Strain, SA)	GSE9960
24. Sepsis	GSE19668 (AJ Strain, SA)	GSE28750
25. Sepsis	GSE19668 (AJ Strain, SA)	GSE13015 (GPL6947)
26. Sepsis	GSE19668 (AJ Strain, SA)	GSE13015 (GPL6106)
27. Sepsis	GSE19668 (C57 Strain, SA)	GSE13904
28. Sepsis	GSE19668 (C57 Strain, SA)	GSE9960
29. Sepsis	GSE19668 (C57 Strain, SA)	GSE28750
30. Sepsis	GSE19668 (C57 Strain, SA)	GSE13015 (GPL6947)
31. Sepsis	GSE19668 (C57 Strain, SA)	GSE13015 (GPL6106)
32. Sepsis	GSE19668 (C57 and AJ Strains, SA)	GSE13904
33. Sepsis	GSE19668 (C57 and AJ Strains, SA)	GSE9960
34. Sepsis	GSE19668 (C57 and AJ Strains, SA)	GSE28750
35. Sepsis	GSE19668 (C57 and AJ Strains, SA)	GSE13015 (GPL6947)
36. Sepsis	GSE19668 (C57 and AJ Strains, SA)	GSE13015 (GPL6106)

Baseline correspondence between each mouse model and human dataset was assessed by differential expression analysis and Gene Ontology (GO) pathway enrichment analysis of differentially expressed, homologous mouse and human transcripts. We computed the precision and recall of the DEGs and pathways with respect to correspondence between mouse and human datasets and summarized these quantities using two F-scores. The F-score gave an equal weighting on the correctness of DEG and pathway predictions (precision) and how comprehensive (recall) the predictions were relative to the human-predicted associations. The F-scores of the machine learning model predictions were calculated by comparing the algorithm-predicted human DEGs and pathways to those derived using the true human phenotypes. The mouse predicted DEGs and enriched pathways constituted the baseline performance against which our machine-learning approaches were compared.

**Fig 1:**
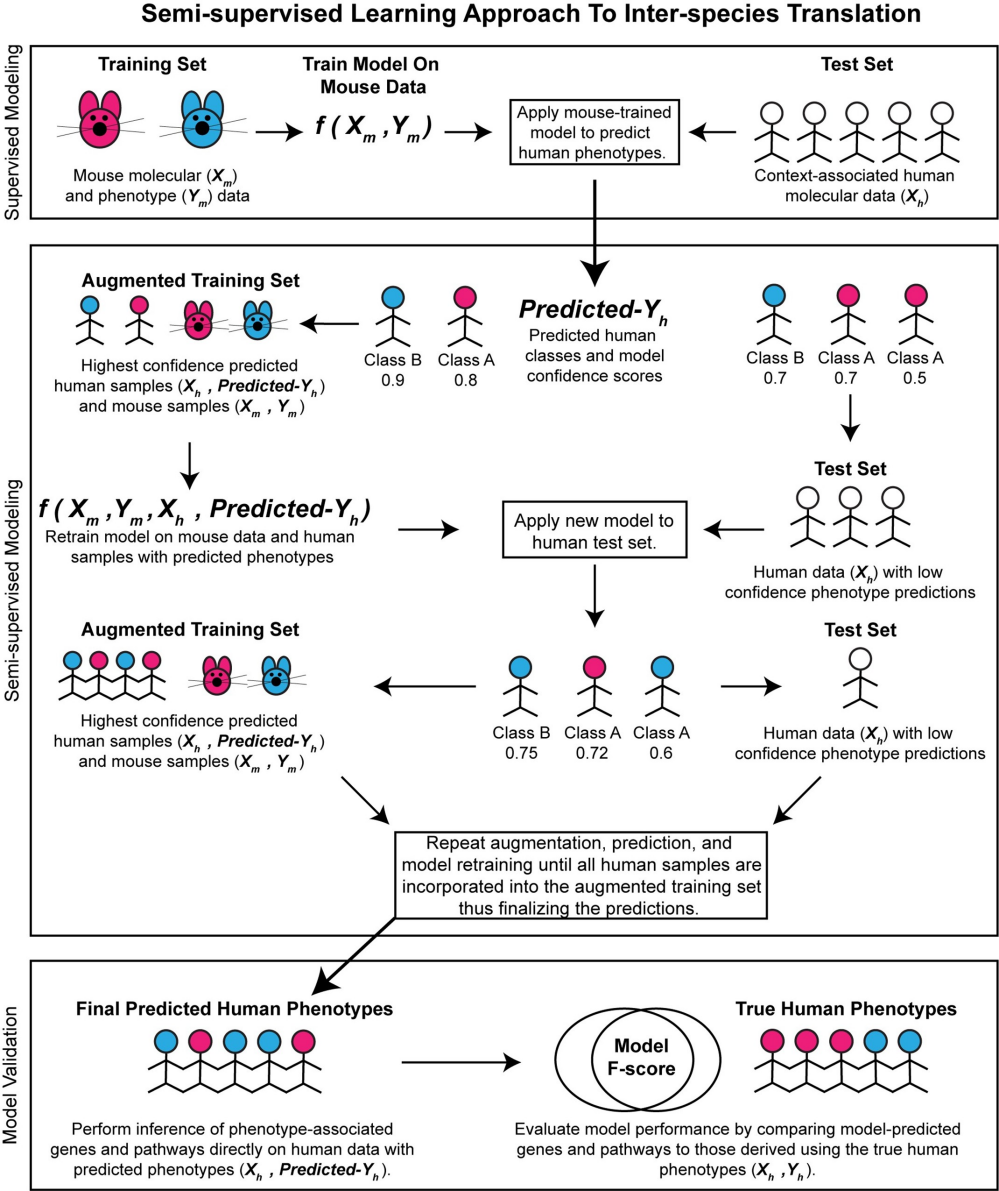
Semi-supervised learning approach to inter-species translation. Semi-supervised learning begins by training an initial supervised model on the mouse data alone and applying the model to a human test data. Human samples with the highest prediction confidence are used to create an augmented training dataset of mouse and human samples with predicted phenotypes. A new model is trained on this augmented training set and applied to reclassify the human samples. Predictions are finalized when all human samples are merged with the training set. Predicted human differentially expressed genes and enriched pathways are validated against genes and pathways identified using the true human phenotypes.

We implemented supervised and semi-supervised versions of k-nearest neighbors (KNN), support vector machine (SVM), random forest (RF), and neural network (NN) algorithms using Lasso or elastic net (EN) regularization as a feature selection method. By exploring a range of machine learning models with different model structure and varying the regularization parameter α, we were able to assess the effect of model structure and feature selection stringency on performance. In supervised models, a machine learning classifier was trained on the mouse dataset and applied to the human test dataset to infer predicted phenotypes from which we inferred human DEGs and enriched pathways. In semi-supervised models, a supervised classifier was initially trained on the mouse data alone to predict the human samples. Following this first step, the predicted human samples with the highest classification confidence were selected to create an augmented mouse-human training set (Fig 1). Retraining with the predicted human samples allowed us to humanize the new classifier using unsupervised information from the human test dataset. The new classifier was then used to reclassify the human samples. This procedure of retraining, prediction, merging predicted human samples with the training set, and dropping the confidence threshold each iteration terminated when the lowered confidence threshold resulted in merging all human samples with the training set. The phenotypes associated with the human samples at this step were taken as the final semi-supervised model prediction from which predicted human DEGs and enriched pathways were inferred. Model DEG and pathway F-scores were computed by comparing the algorithm-predicted DEGs and pathways, using computationally inferred human phenotypes on the human test data, to those identified when using the true phenotypes on the human test data.

### Semi-supervised training of a neural network is the most broadly effective inter-species translation model

We compared the performance of 1,728 machine learning classifiers to the mouse-predicted DEG and pathway associations. Classifier performance was summarized by the area under the receiver operator characteristic curve (AUC) for the accuracy of the predicted human phenotypes and the F-score of predicted human DEGs and pathways. A generalized linear model (GLM) was trained to assess the impact of Lasso/EN regularization α values and the type of machine learning classifier on the AUC and DEG F-score performance metrics. Neither the value of α (p = 0.374), nor the type of machine learning approach (p = 0.874) significantly impacted the AUC (S1 Table). However, both α (p = 0.0000215) and the type of machine learning method (p = 0.000902) significantly impacted the F-score (S2 Table). The significance of the regularization parameter and classifier type for F-score and not AUC suggests that though each model had comparable accuracy, the biological relevance of the predicted phenotypes was significantly influenced by feature selection stringency and machine learning model structure.

**Fig 2:**
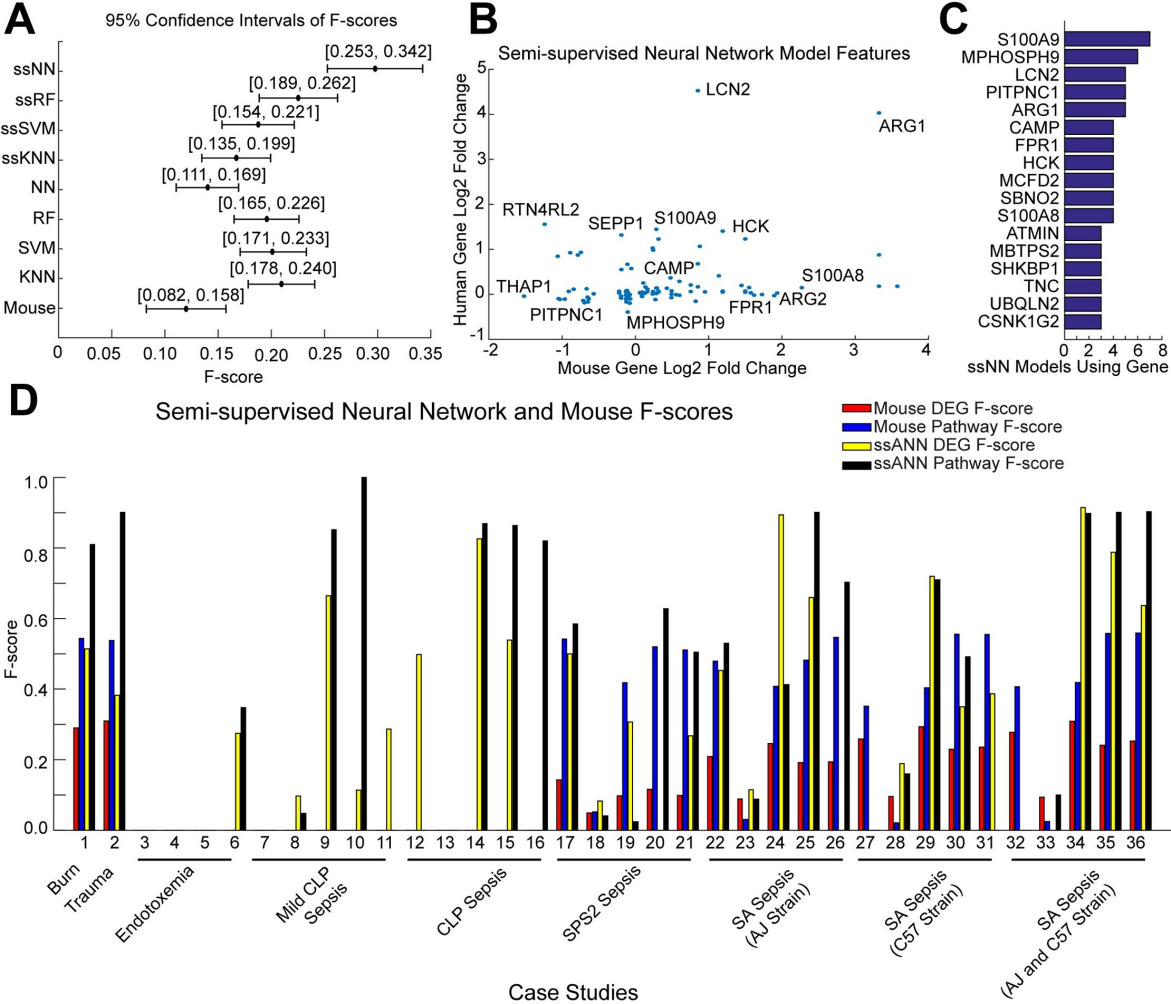
Semi-supervised training of a neural network is the most broadly effective computational translation approach. (A) 95% confidence intervals of the DEG F-scores of each machine learning approach across all regularization parameters and case studies. The average F-score is denoted in the confidence interval. (B) Log2 fold changes of genes included in the ssNN model compared between mouse and human contexts. (C) Frequency of genes included in more than three ssNN models across case studies. (D) Comparison of DEG and pathway F-scores of mouse models and ssNN delineated by case study and disease context.

Since the F-score directly measured the biological relevance of the predictions made by a particular algorithm, we focused on it as the relevant performance metric, emphasizing gaining biological insights over mere numerical predictive capacity. We computed the 95% confidence intervals of the F-scores for each machine learning approach and mouse model across all case studies and regularization parameters (Fig 2A). The overall performance of mouse-derived DEGs for predicting human DEGs was low (F-score 95% CI [0.082, 0.158]) and though many models significantly outperformed the mouse, the F-scores were still somewhat low indicating an imbalance in precision and recall in some case studies. We investigated the role that the experimental design of the mouse cohorts may be contributing to this imbalance using a GLM and found that smaller sample sizes and larger class imbalances in the mouse datasets resulted in significantly lower model F-scores (S3 Table). Though most machine learning models balanced precision and recall, we noted a cluster of models with precision < 0.2 and recall > 0.3 (S1 Fig). All of these could be attributed to case studies in which human dataset GSE9960 was the test dataset (Table 2). Here, the mouse training datasets were comprised of mouse leukocytes and the poor performance of the models suggests that mouse leukocytes are not reflective of human peripheral blood mononuclear cell (PBMC) biology. We retained case studies with GSE9960 to examine whether our models could add translational value despite this inter-tissue mouse and human discrepancy.

The semi-supervised NN (ssNN), semi-supervised RF (ssRF), KNN, SVM, and RF outperformed the mouse model, with similar behavior found for the precision and recall (Fig 2A, S4 and S5 Tables). We found that ssNN F-scores were significantly higher than all other models indicating it was the most successful model (95% CI [0.253, 0.342], p < 0.05). Finally, we examined the performance of the ssNN across all case studies for each setting of the regularization parameter and found Lasso regularization (α = 1.0) had the highest F-score across all case studies (median F-score = 0.281) (S6 Table). Based upon the GLM, F-scores, and performance at each value of α, we concluded that the ssNN with Lasso regularization was the most broadly effective approach for prediction of human DEGs.

Having identified the ssNN as the most broadly effective model, we examined the genes selected in the semi-supervised training procedure (Fig 2B and 2C, S7 Table). Most of the genes selected by the ssNN were not concordantly differentially expressed in mouse and human contexts (Fig 2B). The genes most frequently included in the ssNN models tended to have either strong differential regulation in the human context alone (e.g LCN2) or be among those genes that exhibit concordant differential expression in both mouse and human contexts (e.g. ARG1) (Fig 2C). Recall that the semi-supervised training procedure begins with a model and features informed only by the mouse training dataset, demonstrated by the cluster of genes exhibiting large mouse fold changes. That these genes have correspondingly small human fold changes suggests that the neural network is responsive to the addition of predicted human samples in the training procedure and is able to prioritize those genes that are relevant to the human context and ignore those relevant only in the mouse context (Fig 2B and 2C).

We next compared the DEGs and pathways predicted by the ssNN and mouse models in each case study (Fig 2D, S8 Table). In most cases, the mouse pathway F-score is higher than the DEG F-score indicating that the mouse models considered here are more predictive of human pathway function than differential expression events (Fig 2D). The correspondence between the enriched pathways identified by mouse models and human *in vivo* contexts was relatively consistent across disease indications, (Fig 2D), suggesting that mouse models of inflammatory pathologies recapitulate similar proportions of human *in vivo* molecular biology across indications independent of disease etiology complexity.

Notable exceptions to this pattern of mouse-human pathway correspondence were the endotoxemia and cecal ligation and puncture (CLP) mouse models, none of which, had any corresponding human DEGs at permissive statistical thresholds (WMW p < 0.05, FDR q < 0.25) (Fig 2D). Despite this, in 9 of 14 endotoxemia or CLP mouse cases, the ssNN characterized a large proportion of human sepsis biology despite being trained on nonrepresentative mouse models (Fig 2D). Similarly, in 5 of 6 cases where the human PBMC dataset was the test dataset and mouse leukocyte gene expression was the training set, the ssNN equaled or surpassed the mouse. These results indicate that the semi-supervised approach provided substantial benefit when mouse models, such as CLP-driven sepsis and LPS stimulated endotoxemia, did not recapitulate molecular features of human disease biology.

In total, the ssNN predicted an equal or greater proportion of human enriched pathways in 29 of 36 case studies (Fig 2D). In the other cases, the mouse models of *Streptococcus Pneumoniae Serotype* 2 (SPS2) *and Staphylococcus Aureus* (SA) driven sepsis outperformed the ssNN in particular human cohorts. A single human sepsis dataset, GSE13015, where many of the patients had other infections, was implicated in 3 of these 7 case studies [9]. This suggests that the C57 strain mouse with an SA or SPS2-driven sepsis is an unusually satisfactory direct model for human sepsis with other infectious complications. The ssNN may have failed to outperform the mouse in these cases due to the heterogeneity of infections in the human cohort, an interpretation supported by the fact that the ssNN outperforms the combined mouse cohort by a wide margin when the AJ and C57 mouse models are combined into a single training cohort (Fig 2D). Therefore, when predicting biological associations in a heterogeneous human cohort, the ssNN performs better when trained on a heterogeneous mouse cohort.

### The semi supervised neural network improves comprehensiveness of translating human *in vivo* pathways in sepsis

**Fig 3:**
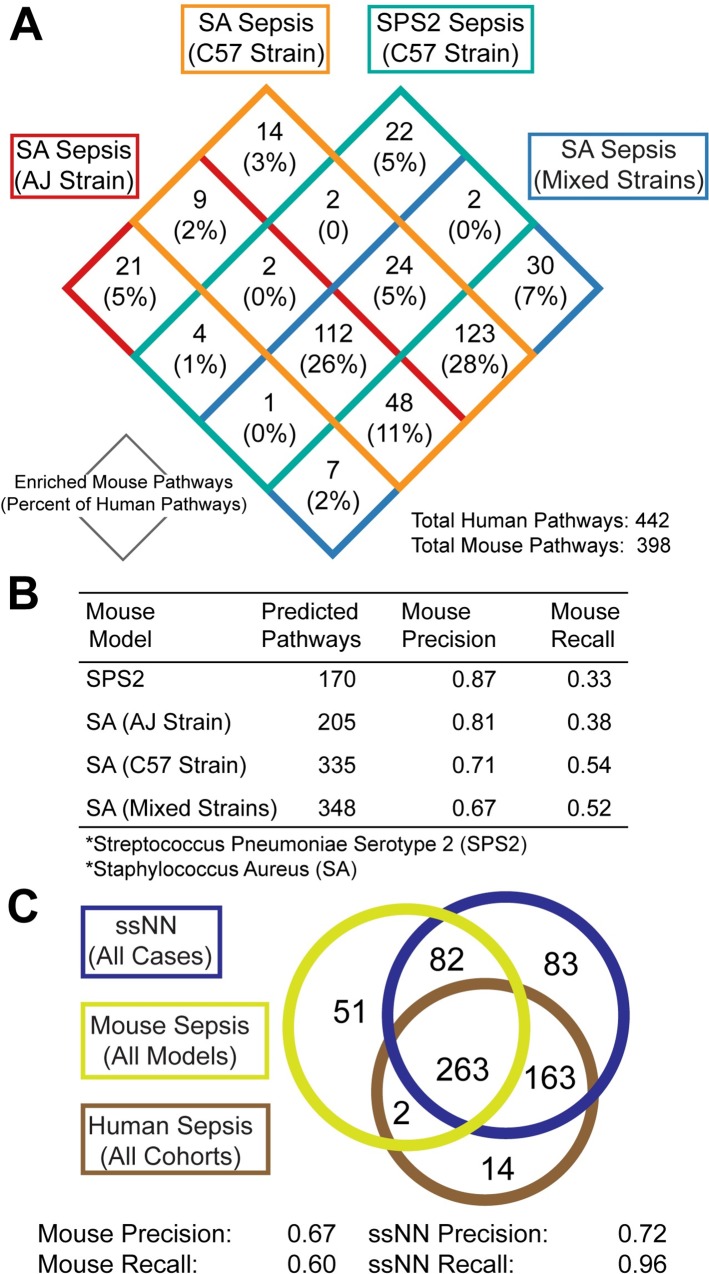
Assessing the correspondence of pathways implicated by mouse models or the ssNN to human *in vivo* sepsis. (A) Comparison of signaling pathways enriched in different strains and mouse models of sepsis and proportion of these pathways enriched in human sepsis. (B) Precision and recall of each mouse model’s predicted human *in vivo* signaling pathways by strain and type of mouse model. (C) Comparison of signaling pathway predictions by any sepsis mouse model, ssNN, and human *in vivo* sepsis.

This diversity of sepsis mouse models in our cohort made it possible to assess the correspondence of different protocols for generating sepsis mouse models to the human disease context. While CLP mouse models failed to identify any DEGs, the SPS2 and SA sepsis mouse models were both partially predictive of DEGs and pathways in human sepsis cohorts. The SA mouse sepsis cohort was comprised of two mouse strains, the highly susceptible A/J mouse strain and the somewhat resistant C57BL/6J strain [8]. We were therefore able to compare four cohorts of sepsis models (SPS2- C57BL/6J, SA-A/J, SA-C57BL6J, and SA-mixed (A/J and C57BL6J)) in order to identify the most representative mouse models of clinical sepsis. Since pathway predictions had a greater correspondence to human sepsis than DEGs alone, we compared the pathway associations derived from each sepsis mouse model to one another to identify common and distinguishing features of each model (Fig 3A). In total, 442 pathways and processes were enriched across all human sepsis cohorts and multiple mouse sepsis models correctly predicted subsets of these pathways. All mouse models and strains correctly identified a set of 112 pathways including signaling by FGFR1, FGFR2, FGFR3, and FGFR4, and MAPK1 signaling (S9 Table). This pathway signature of human sepsis appears to be highly reproducible in multiple mouse sepsis models, rendering it a stable signature for assessing therapeutic interventions and benchmarking mouse sepsis models against human data.

Examining mouse sepsis model F-scores by component precision and recall revealed that while aggregating predictions across multiple mouse models improves the coverage of human sepsis pathway predicted, it simultaneously degrades the precision of these pathway signatures and ultimately only accounts for half of the totality of human *in vivo* sepsis signaling (Fig 3B). This contrasts with our finding that increasing the heterogeneity of the mouse cohort improved the predictive power of the ssNN suggesting that a heterogeneous mouse cohort contains latent features that the ssNN detects and incorporates into its predictions of human *in vivo* pathways. Therefore, a key limitation of these sepsis mouse models appears to be that they lack in depth and correspondence of biological functions to the processes of human *in vivo* sepsis and that the ssNN is able to recover this missing information through integration with human datasets.

We then compared the combined pathway predictions of all mouse sepsis models to the predictions of the ssNN across all sepsis cases to assess correspondence with human *in vivo* sepsis pathway signatures (Fig 3C). The mouse sepsis models confirmed two pathways that the ssNN missed: the CD28-dependent VAV1 pathway and the oxidative stress induced senescence pathway. The oxidative stress senescence pathway was implicated by both of the SA mouse models in isolation, but not the mixed cohort, while the CD28-dependent VAV1 pathway was specifically implicated in the C57 strain. Use of a CD28 mimetic peptide has been shown to increase survival in gram-negative and polymicrobial models of mouse sepsis and has been explored as a therapeutic option for human sepsis [21]. Though the mouse model identified two pathways missed by the ssNN, the ssNN performed with comparable precision to the mouse models overall (precision = 0.72) and recovered a strikingly higher proportion of *in vivo* human sepsis pathways (recall = 0.96) (Fig 3C). Furthermore, the ssNN recovered a set of 163 pathways enriched in human sepsis *in vivo* that were not identified in any mouse models of sepsis (S10 Table). These pathways included thrombin signaling, TGFβ signaling, as well as several RNA transcriptional and post-translational modification-based pathways (S10 Table) that all mouse models of sepsis lacked. Both thrombin and TGFβ signaling have been shown to play key roles in the pathology of sepsis and have been investigated for therapeutic and prognostic applications in sepsis [22, 23] [24]. This result suggests that combining context-associated human data with mouse disease model data recovers important aspects of human *in vivo* signaling.

## Discussion

The lack of fidelity of mouse models for representing complex human biology is one of the most pressing challenges in biomedical science. Failures of inter-species translation are likely driven by a combination of evolutionary factors, experimental design limitations, and the challenges of comparing biological function between species and tissues [25–27]. It is well known that particular features exist that translate well between model systems and humans, particularly at the level of pathway function [28, 29]. However, a key methodological issue in inter-species translation is to consider what will be knowable in prospective translation of a model system experiment, that pre-selection of translatable features is often not possible. In this study we demonstrate that semi-supervised training of a neural network is a powerful approach to inter-species translation and show that successful translation is dependent upon the computational method, the model system-to-human tissue pairings, and the experimental design of the model systems studies. The low pathway recall in the sepsis mouse models demonstrates that there are human disease-associated biological functions simply not present in mouse disease biology. Despite this intrinsic limitation of the mouse, our semi-supervised learning approach prospectively discovers mouse features predictive of human biology, offering a valuable tool for inter-species molecular translation.

**Fig 4:**
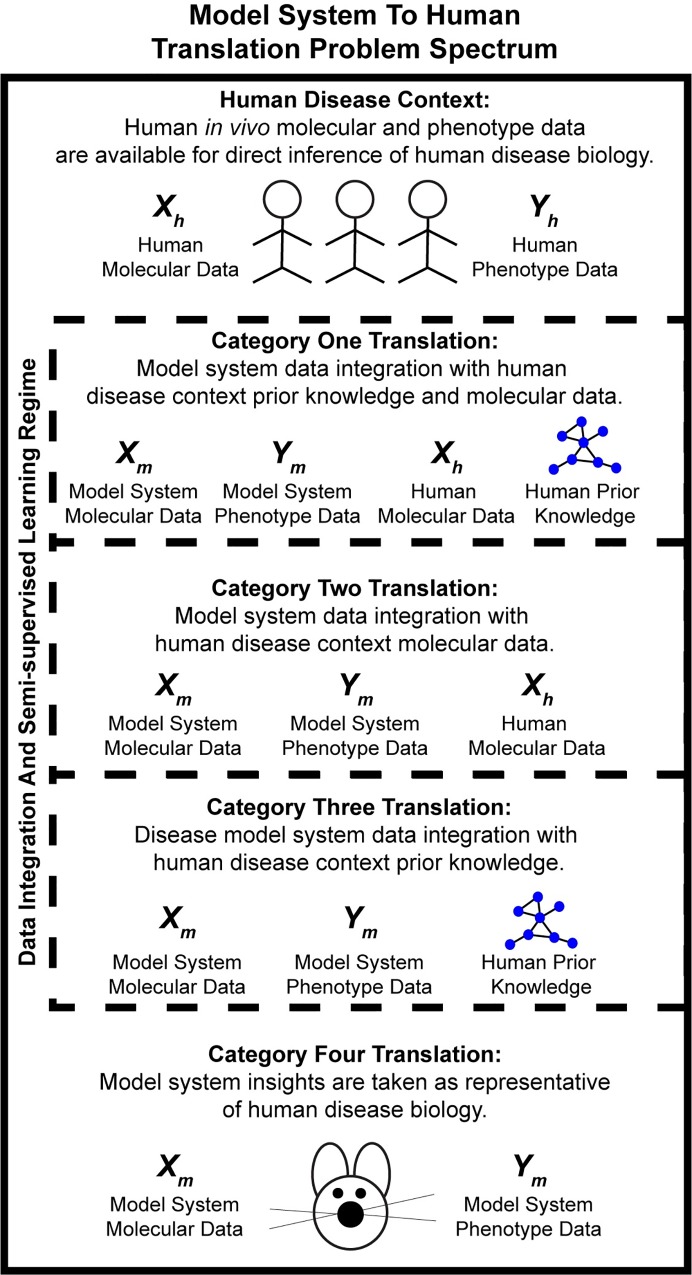
Delineation of four categories of translation problems, those of generalizing insights from model systems to human *in vivo* contexts. The coverage and resolution of the available data determines the category. Categories one through three are potentially approachable by data integration of model system and human datasets and semi-supervised learning approaches.

The ideal case for characterizing the biology of human disorders would be the availability of comprehensive human phenotype and molecular data from clinical cohorts. However, since novel perturbations to the disease system cannot be studied in the human *in vivo* context outside of a clinical trial, mouse disease model systems and emerging human *in* vitro model systems will continue to play an important role in biomedical research. It is in this context that we propose a delineation of four categories of *Translation Problems*, those of generalizing insights from model systems to human *in vivo* contexts (Fig 4). The most challenging case is when only model system molecular and phenotype data are available (Category 4), where a large proportion of biomedical research falls. If human-based prior knowledge, such as candidate genes or clinical observations, is available to integrate with model system data, then generalization can be characterized as a Category 3 problem. In Category 2 problems, condition-specific human molecular data is available to combine with model system molecular and outcome data to characterize human biology. Inferences from solving Category 2 problems can be further refined with human-based prior knowledge in a Category 1 problem.

Within this framework, our efforts here are best viewed as an approach to Category 2 translation problems in which we show that ssNN modeling provides a framework for integration of high throughput, high-coverage datasets from model system and human contexts for molecular translation. However, different categories of translation problems have datasets with different properties and will likely require alternative computational methods. In a recent crowd-sourced competition, a series of challenges were posed for translating molecular and pathway responses between rat and human *in vitro* models. No computational methods were broadly effective in across challenge events and it appears that none of the competitors employed semi-supervised machine learning approaches [30, 31]. This finding supports our delineation of *Translation Problems* into different categories defined by the coverage and resolution of the data available for model training. Other computational translation efforts often use information about how genes change between experimental groups in both model system and human contexts [32, 33]. A key advantage to our approach to inter-species translation is that information about gene regulation in the human context is not required for successful modeling.

The driving principle of semi-supervised learning (transfer learning) is that combining information from multiple domains can enhance model performance. In these applications, a set of training data (X_train_ and Y_train_) are integrated with a context-related dataset (X_context_) to improve the performance of the algorithm in an approach known as inductive transfer learning. Our approach is an example of transductive transfer learning, where X_context_ = X_test_, and the test dataset is incorporated into the algorithm training procedure in an unsupervised manner. Examining machine learning models with different structures allowed us to assess whether different model structures resulted in better performance and how responsive different models structures were to a semi-supervised training procedure. In the case of KNN and SVM models, the human samples were classified by distances in mouse gene expression feature space, a model structure we found did not gain in performance with semi-supervised training. By contrast, the NN and RF improved in performance with semi-supervised training suggesting that these approaches are more responsive to reweighting model features by incorporating unsupervised human information. Although the NN ended up being the most biologically successful model, direct interpretation of NN model weights and neurons remains challenging. Here, we use the NN as a prediction-only model and derive biological insights in downstream analyses, though as NN interpretability methods advance it may be possible to gain additional biological insights by direct interpretation of the NN model structure.

Despite advances in the fidelity of model system biology to human contexts, generalizability of findings of model system experiments will continue to be a key issue in both basic biology and translational science research [34, 35]. Whenever the model system data alone forms the basis of inference, whether through direct interpretation or indirectly through a computational description of the model system’s biology, key aspects of human biology are likely to be overlooked or misrepresented. Semi-supervised learning approaches that neither aim for a generalizable computational model nor rely on the model system training data alone, recover more relevant human *in vivo* biology as a downstream consequence of creating good predictions of human phenotype for a specific patient cohort. This conceptual shift from direct interpretations of model system data to the indirect generalization of model system biology through integration with human data in semi-supervised learning framework has the potential to aid in successful translation of preclinical insights to patients.

## Materials and methods

### Dataset collection and processing

Datasets were obtained from Gene Expression Omnibus [36] and selected based on their inclusion in two studies comparing mouse and human genomic responses [1, 2]. Since we used the human datasets as test datasets and the mouse datasets as training datasets for machine learning applications, we applied the additional criteria that phenotypes and tissues of origin were comparable between mouse model and human *in vivo* datasets to ensure comparable training and test cases for algorithm performance comparison. Based on these criteria, we excluded the acute respiratory distress syndrome and acute infection datasets, and mouse splenocyte samples from GSE7404, GSE5663 antibiotic treated sepsis mice spleen samples, and GSE26472 mouse liver and lung samples. The final cohort consisted of 6 mouse cohorts and 7 human cohorts (Table 1). Mouse array probe identifiers were converted to gene symbols and mapped to homologous human genes using the mouse genome informatics database [37, 38]. If multiple diseases or microarray platforms were used in a dataset, the dataset was partitioned by disease type and array platform to create multiple case studies, resulting in 36 case studies (Table 2). Duplicate genes in each dataset in each case study were removed by retaining those genes with the maximum average expression across all samples. Datasets were z-scored by gene.

### Supervised and semi-supervised classification models

We implemented supervised and semi-supervised versions of the k-nearest neighbors (KNN), support vector machine (SVM), random forest (RF), and neural network (NN) algorithms. Simulations showed that three neighbors were sufficient for training the KNN models (data not shown). Simulations from 10 to 1000 decision trees showed that 50 decision trees were sufficient for training the RF (data not shown). The NN was a feed-forward neural network with three layers. The input layer consisted of one node for each feature, the output layer consisted of two nodes, one for each class, and the hidden layer consisted of the average of the number of input and output nodes rounded up to the nearest integer. NN synapse weights were computed using scaled conjugate gradient backpropagation.

Prior to model training, we performed feature selection with either Lasso or elastic net (EN) regularization. Different values of the regularization parameter α were examined to assess the impact of varying the number of features selected for training the supervised and semi-supervised classifiers (α = 1.0, 0.9, 0.7, 0.5, 0.3, 0.1). In the case of supervised classification models, Lasso and EN regularization underwent 10-fold cross validation (leave one out cross validation for mouse endotoxemia dataset GSE5663) to learn a set of features. These features were then used to train a supervised classifier (KNN, SVM, RF, or NN) on the mouse dataset. The supervised classification model was then applied to the human dataset for that particular case study to infer predicted human phenotypes. In the case of semi-supervised models, feature selection was performed on the mouse dataset in the same manner as supervised models. These features were then used to train an initial supervised classification model on the mouse data alone to predict the human samples’ phenotypes. Following this initial training and prediction step, the human samples with the highest 10% of confidence scores on their predicted phenotypes were combined with the mouse dataset to create a new augmented training set. In the second iteration, feature selection and model training proceeded using this training set of mouse and human samples. All human samples in the test set were re-classified and the confidence score threshold of inclusion was dropped by 10%. Feature selection, model retraining, classification, and training set augmentation continued until all human samples were incorporated into the training set. Since NN training is inherently stochastic, we specified that the semi-supervised NN would proceed to the second iteration only if more than one human sample was classified into each class. If this condition as not met after 50 training iterations, the semi-supervised NN proceeded with further training and prediction iterations on the human dataset using an initial model that did not have human predicted phenotypes in both classes.

### Model performance assessment

Classification models were evaluated by their ability to discriminate between human phenotypes and by the extent to which analyzing the human molecular data using the predicted human phenotypes implicated the same genes as using the true human phenotypes. Classification performance was assessed by the area under the receiver operating characteristic curve (AUC) for the test set of human samples. Differential expression analysis was performed on the homologous mouse and human genes using the phenotypes from the original datasets to identify differentially expressed mouse and human genes. Following model prediction, differential expression analysis was then performed on the human dataset using the predicted phenotypes. Differential expression was assessed by the Wilcoxon-Mann-Whitney (WMW) test with Benjamini Hochberg False Discovery Rate (FDR) correction (significance: WMW p < 0.05 and FDR q < 0.25). GO enrichment was performed on all DEGs in each case study, for the human data, mouse data, and human data with predicted phenotypes using the Reactome pathway database annotation option in GO [39] [40, 41].

A DEG or enriched pathway identified in the mouse model was considered a true positive (TP) if that gene or pathway was also implicated in the human data analyzed using the true phenotypes. False negatives (FN) were DEGs or enriched pathways implicated in the human data, but not implicated by the mouse model. False positives (FP) were DEGs or pathways implicated in the mouse but not in the human data. DEGs and pathways identified using the predicted human phenotypes generated machine learning approaches were considered TP, FP, and FN by their correspondence to the DEGs and pathways implicated in human data analyzed using the true phenotypes. We computed the precision and recall for the DEGs predicted by the mouse model and machine learning classifiers and aggregated these into an F-score for each prediction modality. Enriched pathway precision, recall, and F-scores were analogously computed for TP, FP, and FN predicted pathways from the mouse model and machine learning classifiers.

### Code availability

All analyses were implemented in MATLAB 2016b. KNN, SVM, and RF functions were implemented using the fitcknn, fitcsvm, and TreeBagger functions respectively. Neural networks were implemented using the MATLAB Neural Network Toolbox. Semi-supervised functions are deposited at: https://www.mathworks.com/matlabcentral/fileexchange/69718-semi-supervised-learning-functions

## Supporting information

S1 Table Generalized linear model coefficients for the effect of machine learning model type and elastic net parameter on the AUC performance of machine learning classifiers. (XLSX)

S2 Table Generalized linear model coefficients for the effect of machine learning model type and elastic net parameter on the F-score performance of machine learning classifiers. (XLSX)

S3 Table Generalized linear model coefficients for the effect of machine learning model type, elastic net parameter, and mouse cohort experimental design (sample size and class imbalance) as predictors of F-score performance of machine learning classifiers. (XLSX)

S4 Table 95% confidence intervals of precision and recall of machine learning methods. (XLSX)

S5 Table Significance of F-score performance differences between machine learning methods. (XLSX)

S6 Table Median F-scores of machine learning method by regularization parameter. (XLSX)

S7 Table Genes included in the final ssNN models. (XLSX)

S8 Table Summary of the number of DEGs and enriched pathways associated with each human patient cohort, mouse model cohort, and ssNN predicted associations. (XLSX)

S9 Table Reactome pathways enriched in human sepsis in vivo and consistently recovered by the SA and SPS2 sepsis mouse models. (XLSX)

S10 Table Reactome pathways enriched in human sepsis in vivo, missed by both the SA and SPS2 sepsis mouse models, but recovered by the ssNN. (XLSX)

S1 Fig All machine learning model precision and recall statistics. (TIF)
