# Supplementary figures and images for: Computational translation of genomic responses from experimental model systems to humans
Source: PLoS Comput Biol. 2019 Jan 10;15(1):e1006286. doi: 10.1371/journal.pcbi.1006286 (PMC6343937; doi:10.1371/journal.pcbi.1006286)

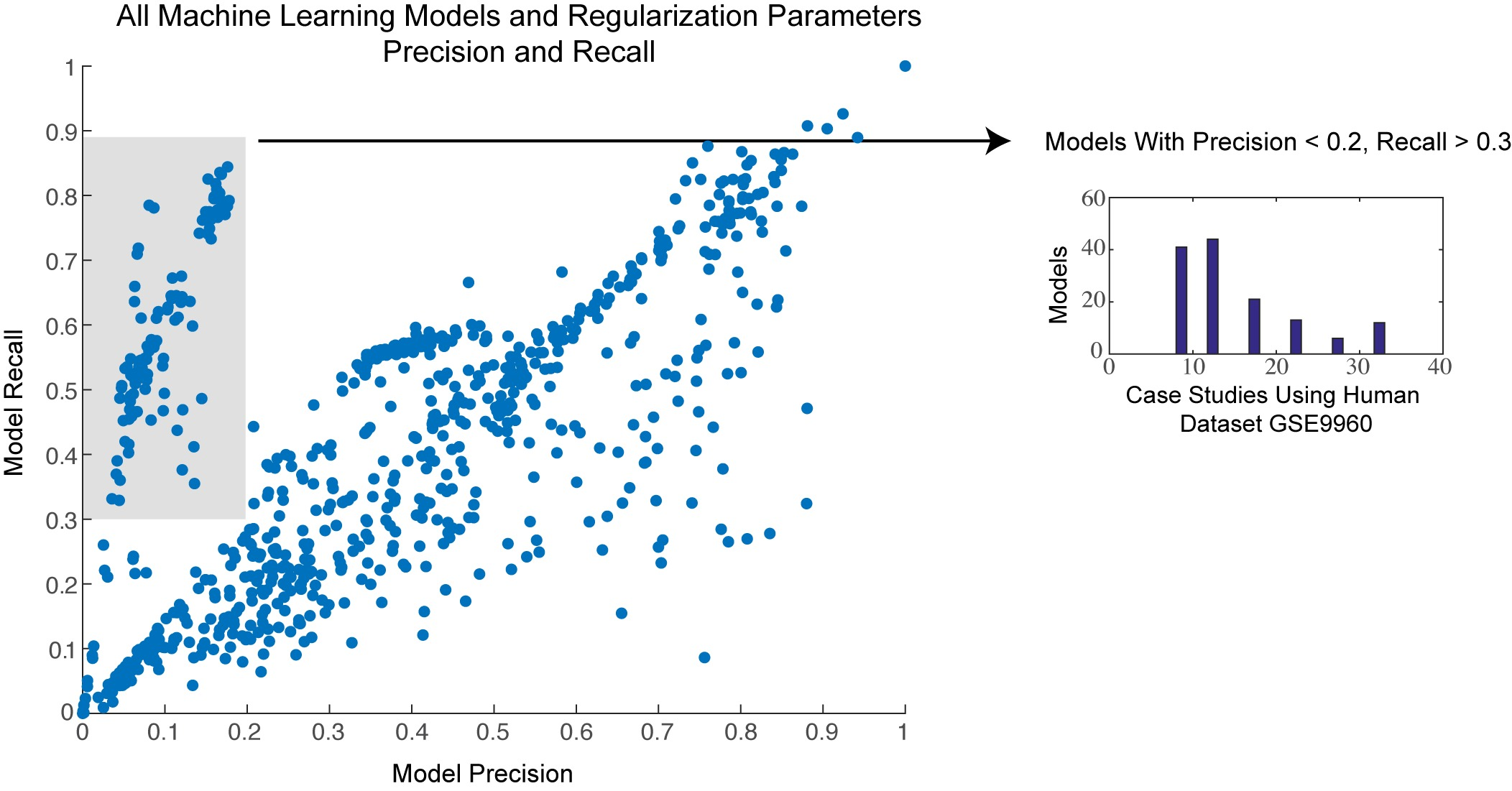

Supplement: S1 Fig — (TIF) [file pcbi.1006286.s011.tif]
